# Effectiveness of ‘Mois sans tabac 2016’: A French social marketing campaign against smoking

**DOI:** 10.18332/tid/139028

**Published:** 2021-07-19

**Authors:** Romain Guignard, Raphaël Andler, Jean-Baptiste Richard, Anne Pasquereau, Guillemette Quatremère, Pierre Arwidson, Karine Gallopel-Morvan, Viêt Nguyen-Thanh

**Affiliations:** 1 Prevention and Health Promotion Department, Santé publique France, the French National Public Health Agency, Saint-Maurice, France; 2 École des Hautes Études en Santé Publique, Rennes, France

**Keywords:** tobacco, smoking cessation, quit attempt, social marketing intervention, effectiveness

## Abstract

**INTRODUCTION:**

In October 2016, the first edition of *Mois sans tabac* (Tobacco-Free Month) was launched, a campaign which had invited French smokers to challenge themselves to quit smoking for the whole month of November. We aimed to study the effectiveness of this social marketing intervention on quit attempts (QA) in the general French population, and to study possible differences according to sociodemographic characteristics.

**METHODS:**

This study used data from the 2017 Health Barometer survey, a random survey conducted by telephone on 25319 individuals. It included 6341 respondents who reported that they were daily smokers when the *Mois sans tabac* campaign was launched in 2016. The association between self-declared exposure to the campaign and making a QA has been studied using multivariate logistic regressions.

**RESULTS:**

Exposure to the 2016 *Mois sans tabac* campaign is associated with a QA lasting at least 24 hours in the final quarter of 2016 (AOR=1.32; 95% CI: 1.07– 1.63, p<0.01), with a QA lasting at least 30 days (AOR=1.95; 95% CI: 1.31–2.91, p<0.001), and being abstinent at the time of the interview in 2017 (AOR=2.39; 95% CI: 1.37–4.15, p<0.01). A dose-effect relationship is observed between the frequency of exposure to the campaign and QA, which is mostly explained by the number of sources of exposure (television, radio, posters, the press, the internet and social networks). Although certain priority groups (e.g. manual workers, the unemployed) had poorer recall of the campaign than other groups, the impact of self-reported exposure to the campaign on QA in unemployed people or those with less than high school educational level appears to have been greater.

**CONCLUSIONS:**

These analyses suggest the effectiveness of the 2016 *Mois sans tabac* intervention, in a context of strengthening public tobacco control policies in France, which may have contributed to the drop in smoking observed between 2016 and 2019.

## INTRODUCTION

Tobacco use kills over seven million people every year worldwide. In Europe, it is responsible for 16% of all deaths in adults^1^. The WHO Framework Convention on Tobacco Control (FCTC) is a response to this epidemic, providing various support tools to develop effective tobacco control policies^2^. One of these tools are mass-media campaigns, as proposed in Article 12 of the FCTC, which calls for the use of all available communication tools to promote and strengthen public awareness of tobacco-control issues. In general, massmedia campaigns to encourage smoking cessation are effective as part of comprehensive tobacco control programmes^3-8^. Another tool proposed in Article 14 of the FCTC is the design and implementation of effective programs aimed at promoting the cessation of tobacco use and providing treatment of tobacco dependence and counselling services in a variety of organizations (i.e. educational institutions, healthcare facilities, workplaces, etc.).

In 2016, Santé publique France, the French National Public Health agency, launched the first edition of a large-scale annual social marketing campaign to create a 30-day quit attempt (QA) challenge for smokers in the month of November, named *Mois sans tabac* (Tobacco-Free Month). This intervention was inspired by the British *Stoptober* campaign, which led to 350000 additional QA (95% CI: 80000–630000) in its first edition in October 2012^9^, and used a new social marketing approach (branding, call to behavior change, promotion of cessation services etc.) for the promotion of smoking cessation in France^10^.

Although many studies have evaluated the effectiveness of anti-smoking campaigns focusing on health hazards and negative emotional messages^4,6^, few have investigated the impact of interventions based on positive and encouraging messages^9,11,12^. Furthermore, most published articles only deal with the evaluation of mass-media campaigns^9,13,14^. Little research has been conducted on the evaluation of global social marketing interventions that include a media component^15^.

This article aimed to document the effectiveness of *Mois sans tabac* 2016 edition on QA, in the wider context of France’s tobacco control program, and to study possible differences in QA according to sociodemographic characteristics.

## METHODS

### Description of the intervention

The goal of *Mois sans tabac* is to encourage smokers to quit smoking for 30 days. Smoking cessation withdrawal symptoms have been shown to dramatically decrease within this timeframe, leading to a five-fold greater chance of permanent quitting^16^. The campaign especially targets smokers aged 20–49 years, as this is the sub-population with the highest smoking prevalence in France, and smokers with a low socioeconomic status, as social inequalities in health related to smoking are particularly present in France^17^.

The intervention combines multi-channel communication (television, radio, posters, internet, social networks), free smoking cessation support services (through a telephone hotline ‘3989’, a dedicated website *tabac-info-service.fr*, an e-coaching application for mobile phones, and a smoking cessation support kit), as well as local actions to raise awareness about and support for smoking cessation (overseen by regional health agencies) in healthcare establishments, the workplace, and other sites.

The *Mois sans tabac* campaign comprises 3 phases over 3 months:

- September: promotion of the campaign to healthcare professionals (letters, advertisements in the professional press, digital content, etc.)
- October: general public media-based recruitment campaign, aimed at encouraging smokers to make a QA and to register on the site https://mois-sanstabac.tabac-info-service.fr
- November: the challenge itself, with support being given to smokers who make a QA.

In 2016, 180113 people registered online for the campaign. A more complete description of the social marketing campaign is available elsewhere^10,18^.

### Data collection

Health Barometer is a large annual random phone survey on health behaviors and perceptions of people living in France set up by Santé publique France^19^. The 2017 fieldwork was conducted by the survey firm Ipsos between 5 January and 18 July on 25319 people aged 18–75 years. The participation rate was 48.5%. The present study and analyses are based on data from the 2017 edition, which collected data on multiple topics including substance use (tobacco, alcohol, illicit drugs), mental health, physical activity, and vaccination.

### Variables

#### Smoking status

Smoking status at the time of the questionnaire (i.e. 2017) was obtained with three questions: ‘Do you smoke tobacco, even if only occasionally?’. Those who answered ‘yes’ to the first question were then asked: ‘Do you smoke every day?’ and ‘How many cigarettes (manufactured or roll-your-own) do you smoke on average?’. Respondents who reported either that they smoked every day (second question) or that they smoked a certain number of cigarettes per day (third question) were classified as daily smokers in 2017.

Smokers reporting not smoking every day (second question) and reporting a number of cigarettes per week, month or year (third question) were not included in the analysis, even though their average consumption was at least one cigarette per day.

Non-smokers (i.e. those replying ‘no’ to the first question) were asked: ‘In your lifetime, have you ever tried smoking tobacco?’ and if they answered ‘yes’, they were then asked, ‘Did you smoke just once or twice, just to try it, or occasionally or daily for less than 6 months, or daily for more than 6 months?’. Former daily smokers were asked when exactly they quit smoking. Those who declared they had quit 12 months or less prior to the survey were asked the month and year they quit. These data enabled us to recode ex-smokers who quit smoking after 1 October 2016 as smokers at the time of the launch of the first *Mois sans tabac* campaign in 2016.

In the remainder of this article, the analyzed study sample corresponds to people who smoked daily at the launch of *Mois sans tabac* in 2016 (n=6341), comprising either self-reported daily smokers in 2017 (n=5968), or ex-daily smokers in 2017 who quit smoking after 1 October 2016 (n=373). For the present study, we hypothesized that the proportion of people who started smoking after *Mois sans tabac* 2016 among daily smokers at the time of the survey was negligible, given that our study sample included those aged 18–75 years.

Among smokers in 2017, tobacco dependence was measured using the Heaviness of Smoking Index (HSI)^20^, and two questions asked respondents to indicate on a scale 0–10 how important they felt smoking cessation was, and on a similar scale their confidence in their own ability to quit.

#### Main outcomes

The present study’s primary dependent variable, was defined *a priori* as a quit attempt (QA) of at least 24 hours in the final quarter of 2016, among daily smokers at the time of the launch of the *Mois sans tabac* campaign.

To measure this, those classified as daily smokers in 2017 were asked: ‘Did you try to quit smoking for at least 24 hours in the last quarter of 2016, i.e. between October and December?’. Former smokers who quit smoking in the final quarter of 2016 were included in the group of those who made a QA. Those who reported a quit date after January 2017, were asked if they had already made a QA in the final quarter of 2016.

Data on the duration of QA were collected. These enabled us to study QA of at least 7 days and at least 30 days. Finally, we estimated the proportion of smokers who made an attempt to quit in the final quarter of 2016 and who were abstinent for at least 7 days at the time of the survey in 2017.

#### Exposure variables

The level of exposure to the *Mois sans tabac* campaign was collected using the questions ‘During the final quarter of 2016, the first *Mois sans tabac* campaign took place. Have you heard about it, whether on TV, on the radio, on the internet, in the press or through another channel?’ (‘ever exposure’). Those who replied ‘yes’ were then asked, ‘How often did you see or hear about the *Mois sans tabac* campaign?’ (1: Several times a day, 2: Once a day, 3: Several times a week, 4: Once a week, 5: Less than once a week). The frequency of exposure was recoded as daily/weekly/ less than weekly/never.

More detailed data on the different sources of exposure were also collected, allowing a number of sources of exposure to be calculated from television, radio, posters, print, internet and social media.

#### Sociodemographic variables

The sociodemographic variables considered in the analyses are sex, age, size of urban area of residence, educational level, professional situation (working; unemployed, looking for a job; inactive), and socioprofessional category, either of the respondents themselves (current professional position for employed persons and last occupation for retired and unemployed respondents) or of the head of the household for other inactive persons (e.g. students, persons with disability).

### Statistical power calculation

The statistical power calculation was based on the following assumptions:

1. A sample size for the 2017 Health Barometer between 15000 and 30000 respondents;
2. A daily smoking prevalence of 28.2% (as observed in the 2014 Health Barometer), which corresponded to a sample of 4230 to 8500 daily smokers for the planned analysis, depending on the final sample size of the Health Barometer;
3. A QA rate of 10% during the final quarter of 2016 in individuals not exposed to the *Mois sans tabac* campaign (as observed for the rate of QA in the final quarter lasting at least 7 days observed in the 2010 Health Barometer^21^, unpublished data); and
4. A self-reported campaign exposure rate of 80% (as observed in the French nationwide *Messages d’adieux* (Farewell Messages) smoking cessation campaign in 2014)^22^.

Based on an estimated sample of 4230 smokers, a difference of 4 percentage points in QA rate could be detected in the group of people exposed to the campaign, using the Casagrande and Pike formula^23^, with an α=0.05 (for a type I error) and 1-β=0.20 (for a type II error). Based on an estimated sample of 8500 smokers, a difference of 2–3 percentage points could be detected.

### Statistical analysis

Bivariate analyses were performed between sociodemographic characteristics, self-reported campaign exposure, QA in the final quarter of 2016, and smoking abstinence in 2017. Differences were tested using Pearson’s chi-squared test with the second-order Rao-Scott correction.

The sociodemographic factors associated with selfreported campaign exposure were estimated using multivariate logistic regression. For each outcome (24-hour QA, 7-day QA, 30-day QA, 7-day abstinence in 2017), three separate logistic regressions were conducted. The associations between ever exposure/ frequency of exposure (see above) and each outcome were established from two separate logistic regressions adjusted for sociodemographic characteristics. The number of sources of exposure (as a continuous variable) was introduced into a third model, adjusted for sex, age, educational level, professional status, socio-professional category, size of urban area of residence and self-reported frequency of exposure to the *Mois sans tabac* campaign. For the primary effectiveness outcome, analyses were stratified according to sociodemographic characteristics and interactions were tested.

To take into account possible bias due to selfreported recall of the campaign that could be linked to respondents’ motivation to quit smoking or quit attempt behavior, sensitivity analyses on the main effectiveness outcome were conducted on the subpopulation of daily smokers in 2017 (94% of the whole sample), by adjusting models for tobacco dependence, the importance they gave to stopping smoking (on a scale 0–10), and their level of confidence in their own ability to quit (on a scale 0–10). These adjustment variables were not available for former smokers at the time of the interview (6% of the whole sample) thus it was not possible to conduct the same analysis for them.

Estimated percentages were first weighted using inverse probability weighting, then adjusted by post-stratification weighting for sex according to age (in 10-year increments), region of residence, size of urban area of residence, number of people in household, and educational level of the resident population in mainland France (Reference population: 2016 National Institute of Statistics and Economic Studies, INSEE, employment survey).

## RESULTS

A description of the sample of daily smokers at the launch of the *Mois sans tabac* campaign in 2016 is shown in Table 1. In the full sample, 54.1% were males, 56.9% were aged 25–49 years, 56.0% had less than high school diploma, 14.9% were unemployed but looking for a job and 31.8% were blue-collar workers. Among smokers at the time of interview in 2017, 43.6% were minimally dependent smokers (HSI=0–1), 38.0% were moderately dependent (HSI=2–3) and 18.4% were highly dependent (HSI =4–6).

**Table 1:** Characteristics of the sample of daily smokers at the time of the launch of the 2016 ‘Mois sans tabac’ campaign (N=6341)

*Characteristics*	*Unweighted n*	*Weighted %*
**Sex**		
Men	3267	54.1
Women	3074	45.9
**Age** (years)		
18–24	711	13.2
25–49	3365	56.9
≥50	2265	29.9
**Educational level**		
<High-school diploma	2786	56.0
High-school diploma	1515	20.9
>High-school diploma	2029	23.1
**Professional status**		
Working	3964	60.3
Unemployed	752	14.9
Inactive	1625	24.9
**Socio-professional category**		
Farmer, craftsperson, trader or business manager (independent professional)	489	8.0
Executive or higher intellectual professional	785	8.9
Intermediate professional	1621	21.7
Employee	1771	29.6
Blue-collar worker	1613	31.8
**Size of area of residence** (inhabitants)		
Rural	1622	22.6
<20000	1126	17.8
20000–99999	802	12.2
100000–199999	390	5.4
≥200000	1497	27.9
Paris area	890	14.0
**Among smokers in 2017**		
**Heaviness of smoking index**		
0–1	2756	43.6
2–3	2228	38.0
4–6	933	18.4
**Importance of quitting smoking** (0 –10), mean ± SD	6.45 ± 2.98	
**Self-confidence in quitting** (0–10), mean ± SD	5.65 ± 2.91	

Executive or higher intellectual professionals include senior executives in civil service, corporate administrative and commercial executives, liberal professionals, third-level teachers/professors, engineers, etc. Intermediate professionals include school teachers, middle-managers, executive officers in the civil service, technicians, etc. Employees include administrative officers in the civil service, administrative or commercial employees, service industry employees, soldiers, etc.

### Self-reported exposure to the campaign and associated factors

Among smokers at the launch of *Mois sans tabac* 2016, 83.3% said in 2017 they had heard of the campaign, more often women (87.7%) than men (79.5%, AOR=1.63; 95% CI: 1.39–1.91, p<0.001); 40.7% said they had heard of it on a daily basis, 32.4% on a weekly basis, while 9.8% had heard of it less than once per week.

Self-reported exposure to the campaign increased with age. Unemployed persons looking for a job (76.3%, AOR=0.67; 95% CI: 0.55–0.82 compared with the employed, p<0.001) and manual workers (80.3%, AOR=0.76; 95% CI: 0.60–0.95 compared with middlemanagers, p<0.05), were less likely to report hearing about the campaign (Table 2).

**Table 2:** Factors associated with ever exposure to ‘Mois sans tabac’, among daily smokers when the campaign was launched in 2016 (N=6341)

*Characteristics*	*%*	*AOR*	*95 % CI*
**Sex**	***		
Men (Ref.)	79.5	1	
Women	87.7	1.63***	1.39–1.91
**Age** (years)	***		
18–24 (Ref.)	78.4	1	
25–49	81.0	1.23	0.98–1.54
≥50	89.7	2.33***	1.81–2.98
**Educational level**			
<High-school diploma (Ref.)	82.6	1	
High-school diploma	84.3	1.15	0.95–1.39
>High-school diploma	84.6	1.08	0.88–1.32
**Professional status**	***		
Working (Ref.)	83.7	1	
Unemployed	76.3	0.67***	0.55–0.82
Inactive	86.4	1.18	0.96–1.45
**Socio-professional category**	***		
Farmer, craftsperson, trader or business manager (independent professional)	78.3	0.57***	0.43–0.75
Executive or higher intellectual professional	82.8	0.73*	0.57–0.94
Intermediate professional (Ref.)	87.3	1	
Employee	85.9	0.90	0.72–1.13
Blue-collar worker	80.3	0.76*	0.60–0.95
**Size of area of residence** (inhabitants)	***		
Rural (Ref.)	88.6	1	
<20000	85.7	0.82	0.65–1.05
20000–99999	79.9	0.67**	0.52–0.86
100000–199999	81.0	0.68*	0.49–0.94
≥200000	82.4	0.70**	0.56–0.87
Paris area	77.3	0.49***	0.39–0.63

AOR: adjusted odds ratio. Ref: reference category in the logistic regression model.

* p<0.05

** p<0.01

*** p<0.001.

Percentages of respondents who recalled Mois sans tabac and p for bivariate analyses between sociodemographic variables and recall of Mois sans tabac, adjusted odds ratios from logistic regression including all variables in the table. Executive or higher intellectual professionals include senior executives in civil service, corporate administrative and commercial executives, liberal professionals, third-level teachers/professors, engineers, etc. Intermediate professionals include school teachers, middle-managers, executive officers in the civil service, technicians, etc. Employees include administrative officers in the civil service, administrative or commercial employees, service industry employees, soldiers, etc.

The sensitivity analysis showed that among daily smokers in 2017, after controlling for tobacco dependence, the importance they gave to quitting, and the level of confidence they had in their own ability to quit, the findings for the sociodemographic variables explored were similar to those observed among respondents who were categorized as smokers at the time of the launch of the campaign in 2016. Moderately dependent smokers (HSI=2–3) were more likely to have heard of the campaign (85.3%, AOR=1.30; 95% CI: 1.09–1.54 compared with minimally dependent smokers HSI=0–1, p<0.01). Respondents’ confidence in their own ability to stop smoking was negatively associated with hearing about the campaign (AOR=0.95; 95% CI: 0.92–0.98 per each additional unit) (Supplementary file, Table S1).

The average number of exposure sources was 2.3. More specifically, 14.9% of smokers reported hearing about *Mois sans tabac* through one media channel, 20.8% two channels, 19.0% three channels, 14.4% four channels, 7.7% five channels, and 3.7% through six channels.

### Quit attempts and smoking status in 2017 according to exposure to the campaign

#### Analysis according to socioeconomic characteristics and ever exposure

Among respondents who were daily smokers at the launch of *Mois sans tabac* 2016, 15.9% made a QA lasting at least 24 hours in the final quarter of 2016, 4.9% made a QA lasting at least 30 days, and 2.8% were abstinent for at least 7 days at the time they were interviewed in 2017 (i.e. 17.8% of those who made a QA in the final quarter of 2016). No significant difference was observed between both sexes. People aged 18–24 years were more likely to have made a QA – irrespective of the duration – but not more likely to be abstinent in 2017. Smokers with an educational level higher than high-school diploma, as well as those belonging to the French socio-professional ‘Executive or higher intellectual profession’ category (which includes senior civil servants, administrative and commercial executives, liberal professionals, third-level teachers/professors among others) were also more likely to have attempted quitting in the last quarter of 2016 and to be abstinent in 2017 (Table 3). Unemployed respondents looking for a job were as likely as employed persons to have tried quitting for at least 24 hours, but after adjusting for other sociodemographic characteristics, they were less likely to have made a QA lasting at least 7 days (AOR=0.71; 95% CI: 0.53–0.97, p<0.05) and to be abstinent in 2017 (AOR=0.54; 95% CI: 0.30–0.96, p<0.05). No association was observed between QA in the final quarter of 2016 or smoking cessation in 2017 and the size of the area of residence (Table 3).

**Table 3:** Factors associated with quit attempts (QA) in the final quarter of 2016 and with smoking abstinence of at least 7 days in 2017, among daily smokers at the time of the launch of the 2016 ‘Mois sans tabac’ campaign: sociodemographic factors, exposure to the campaign, frequency and number of exposure sources (N=6341)

*Characteristics*	*24-hour QA*			*7-day QA*			*30-day QA*			*Smoking abstinence in 2017*		
	*%*	*AOR*	*95% CI*	*%*	*AOR*	*95% CI*	*%*	*AOR*	*95% CI*	*%*	*AOR*	*95 % CI*
**Sex**												
Men (Ref.) (n=3267)	16.4	1		9.7	1		5.1	1		3.0	1	
Women (n=3074)	15.3	0.99	0.86–1.15	8.9	1.03	0.86–1.23	4.6	1.03	0.82–1.31	2.6	0.97	0.72–1.31
**Age** (years)	***			***								
18–24 (Ref.) (n=711)	24.2	1		14.3	1		5.4	1		2.4	1	
25–49 (n=3365)	15.3	0.54***	0.43–0.66	9.1	0.52***	0.40–0.67	5.00	0.65*	0.45–0.93	3.1	1.05	0.63–1.74
≥50 (n=2265)	13.5	0.53***	0.42–0.65	7.6	0.53***	0.40–0.69	4.4	0.70	0.48–1.01	2.5	0.86	0.51–1.45
**Educational level**	***			***			*			**		
<High–school diploma (Ref.) (n=2786)	14.0	1		7.7	1		4.2	1		2.3	1	
High-school diploma (n=1515)	17.3	1.07	0.89–1.28	10.6	1.16	0.92–1.46	5.0	1.10	0.81–1.50	2.6	1.03	0.69–1.54
>High-school diploma 19.4 (n=2029)	19.4	1.31**	1.09–1.58	12.00	1.46**	1.16–1.84	6.3	1.51**	1.12–2.04	4.2	1.50*	1.02–2.20
**Professional status**												
Working (Ref.) (n=3964)	15.5	1		9.6	1		5.3	1		3.2	1	
Unemployed (n=752)	17.4	1.00	0.80–1.24	8.1	0.71*	0.53–0.97	3.9	0.68	0.45–1.02	2.2	0.54*	0.30–0.96
Inactive (n=1625)	16.1	0.91	0.76–1.09	9.4	0.87	0.70–1.08	4.5	0.81	0.61–1.07	2.4	0.81	0.55–1.19
**Socio-professional category**	***			***			***			*		
Farmer, craftsperson, trader or business manager (independent professional) (n=489)	12.4	0.82	0.61–1.11	7.4	0.87	0.60–1.25	3.3	0.94	0.58–1.51	2.1	0.76	0.40–1.45
Executive or higher intellectual professional (n=785)	23.4	1.26*	1.01–1.56	16.7	1.46**	1.13–1.89	9.7	1.57**	1.13–2.19	5.3	1.48	0.97–2.24
Intermediate professional (Ref.) (n=1621)	16.8	1		9.3	1		4.3	1		2.7	1	
Employee (n=1771)	14.4	0.91	0.75–1.11	8.3	0.94	0.74–1.20	4.4	1.01	0.74–1.40	2.6	1.04	0.70–1.56
Blue-collar worker (n=1613)	15.3	0.93	0.75–1.16	8.5	0.92	0.70–1.20	4.7	0.96	0.67–1.38	2.6	0.84	0.52–1.34
**Size of area of residence** (inhabitants)												
Rural (Ref.) (n=1622)	14.3	1		7.9	1		4.8	1		3.1	1	
<20000 (n=1126)	14.4	0.95	0.76–1.18	8.9	1.04	0.80–1.36	4.9	1.01	0.72–1.41	2.5	1.03	0.67–1.58
20000–99999 (n=802)	14.8	1.03	0.81–1.31	9.3	1.21	0.91–1.60	5.1	1.10	0.76–1.58	2.9	1.09	0.68–1.75
100000–199999 (n=390)	17.0	1.00	0.74–1.37	10.3	1.00	0.68–1.47	4.4	0.80	0.47–1.35	2.7	0.69	0.34–1.42
≥200000 (n=1497)	17.4	1.14	0.94–1.39	10.1	1.14	0.90–1.45	4.6	0.88	0.64–1.21	2.3	0.90	0.59–1.35
Paris area (n=890)	18.3	1.03	0.82–1.29	10.5	0.88	0.66–1.18	5.5	0.79	0.54–1.15	4.0	1.01	0.64–1.59
**Had heard of Mois sans tabac^a^**				*			**			***		
No (Ref.) (n=937)	13.6	1		6.8	1		2.9	1		1.1	1	
Yes (n=5404)	16.4	1.32**	1.07–1.63	9.8	1.62***	1.23–2.14	5.3	1.95***	1.31–2.91	3.2	2.39**	1.37–4.15
**Frequency of exposure to Mois sans tabac^a^**							*			**		
Never (Ref.) (n=937)	13.6	1		6.8	1		2.9	1		1.1	1	
Less than weekly (n=610)	14.7	1.02	0.76–1.38	9.2	1.26	0.86–1.86	5.6	1.71	* 1.02–2.89	4.1	2.12*	1.06–4.25
Weekly (n=2057)	17.5	1.32*	1.06–1.66	10.0	1.59**	1.18–2.14	5.3	1.89**	1.24–2.88	3.0	2.33**	1.30–4.17
Daily (n=2561)	16.1	1.43**	1.15–1.79	10.0	1.80***	1.34–2.41	5.2	2.10***	1.38–3.18	3.1	2.50**	1.40–4.44
**Number of exposure sources** (continuous)^b^												
		1.10***	1.04–1.16		1.09**	1.02–1.17		1.13**	1.04–1.23		1.21***	1.09–1.34

AOR: adjusted odds ratio. QA: quit attempt. Ref: reference category in the logistic regression model.

* p<0.05

** p<0.01

*** p<0.001.

Percentages of QA and smoking abstinence in 2017 and p-values for bivariate analyses between sociodemographic variables and quit attempts or smoking abstinence in 2017, adjusted odds ratios from logistic regressions. Executive or higher intellectual professionals include senior executives in civil service, corporate administrative and commercial executives, liberal professionals, third-level teachers/professors, engineers, etc. Intermediate professionals include school teachers, middle-managers, executive officers in the civil service, technicians, etc. Employees include administrative officers in the civil service, administrative or commercial employees, service industry employees, soldiers, etc.

a Results of the logistic model after adjustment for sex, age, educational level, professional status, socio-professional category and size of the area of residence.

b Results of the logistic model after adjustment for sex, age, educational level, professional status, socio-professional category, size of the area of residence and exposure frequency to the Mois sans tabac campaign.

Analyses highlighted an association between selfreported ever exposure to *Mois sans tabac*, assessed in 2017, and: 1) making a QA lasting at least 24 hours in the final quarter of 2016 (AOR=1.32; 95% CI: 1.07–1.63, p<0.01); 2) a QA lasting at least 7 days (AOR=1.62; 95% CI: 1.23–2.14, p<0.001); 3) a QA lasting at least 30 days (AOR=1.95; 95% CI: 1.31–2.91, p<0.001); and 4) abstinence from smoking for at least 7 days at the time of the survey in 2017 (AOR=2.39; 95% CI: 1.37–4.15, p <0.01).

Among daily smokers in 2017, after adjusting for tobacco dependence and their attitudes towards quitting smoking, the association between exposure to the campaign and making a QA lasting at least 24 hours in the final quarter of 2016 remained significant (AOR=1.26; 95% CI: 1.01–1.59, p<0.05). Making a QA was associated with low tobacco dependence (AOR=0.51; 95% CI: 0.43–0.61 for HSI=2–3 and AOR=0.46; 95% CI: 0.35–0.60 for HSI=4–6 compared with HSI=0–1), importance of quitting smoking (AOR=1.14; 95% CI: 1.11–1.18 per each additional unit) and self-confidence in quitting (AOR=1.06; 95% CI: 1.03–1.09) (Supplementary file Table S2).

#### Analysis according to frequency of exposure and number of exposure sources

Analysis by frequency of exposure showed that odds ratios associated with QA increased with frequency of exposure. Accordingly, after adjustment for sociodemographic characteristics, the probability of having made a QA in the final quarter of 2016 or of being abstinent in 2017 was highest for daily exposure to the *Mois sans tabac* campaign, as assessed in 2017. Weekly exposure to the campaign was also associated with making a QA or cessation in 2017, but to a lesser extent (Table 3).

Among smokers in 2017, after adjusting for tobacco dependence and their attitudes towards quitting smoking, only daily exposure to the campaign remained significantly associated with making a QA lasting at least 24 hours in the final quarter of 2016 (AOR=1.39; 95% CI: 1.09–1.77, p<0.01) (Supplementary file Table S 2).

The association between frequency of exposure and QA or smoking cessation was no longer significant after adding the number of sources of exposure to the main models. In contrast, the association between QA or cessation and the number of sources of exposure was significant (24-hour QA: AOR=1.10; 95% CI: 1.04–1.16, p<0.001; 7-day QA: AOR=1.09; 95% CI: 1.02–1.17, p=0.008; 30-day QA: AOR=1.13; 95% CI: 1.04–1.23, p=0.004; abstinent for 7 days in 2017: AOR=1.21; 95% CI: 1.09 –1.34, p<0.001).

#### Interactions between exposure to the campaign and sociodemographic characteristics

There was no statistically significant interaction between exposure to the campaign and sociodemographic characteristics (Table 4). Nevertheless, a stronger association between exposure and a QA lasting at least 24 hours in the final quarter of 2016 was observed for women (AOR=1.65; 95% CI: 1.14–2.37, p<0.01), people aged <50 years (18–24 years: AOR=1.62; 95% CI: 0.97–2.73, not significant; 25–49 years: AOR=1.32; 95% CI: 1.01–1.73, p<0.05), smokers with an education level lower than highschool diploma (AOR=1.64; 95% CI: 1.15–2.33, p<0.01), unemployed respondents looking for a job (AOR=1.85; 95% CI: 1.06–3.23, p<0.05) and the French ‘employees’ socio-professional category (which includes lower level civil service, administrative, commercial and service employees) (AOR=1.64; 95% CI: 1.03–2.61, p<0.05).

**Table 4:** Associations between self-reported exposure to the 2016 ‘Mois sans tabac’ campaign and making a quit attempt lasting at least 24 hours in the final quarter of 2016, among daily smokers at the time of the launch of the campaign, according to sociodemographic characteristics

*Characteristics*	*AOR*	*95% CI*	*p for interaction*
**Sex**			0.122
Men (n=3267)	1.19	0.92–1.53	
Women (n=3074)	1.65**	1.14–2.37	
**Age** (years)			0.471
18–24 (n=711)	1.62	0.97–2.73	
25–49 (n=3365)	1.32*	1.01–1.73	
≥50 (n=2265)	1.15	0.75–1.76	
**Educational level**			0.261
<High-school diploma (n=2786)	1.64**	1.15–2.33	
High-school diploma (n=1515)	1.07	0.71–1.62	
>High-school diploma (n=2029)	1.22	0.87–1.71	
**Professional status**			0.594
Working (n=3964)	1.24	0.96–1.60	
Unemployed (n=752)	1.85*	1.06–3.23	
Inactive (n=1625)	1.29	0.80–2.08	
**Socio-professional category**			0.587
Farmer, craftsperson, trader or business manager (independent professional) (n=489)	1.08	0.54–2.15	
Executive or higher intellectual professional (n=785)	1.33	0.80–2.20	
Intermediate professional (n=1621)	1.43	0.91–2.22	
Employee (n=1771)	1.64*	1.03–2.61	
Blue-collar worker (n=1613)	1.10	0.75–1.60	
**Size of area of residence** (inhabitants)			0.408
Rural (n=1622)	1.35	0.83–2.19	
<20000 (n=1126)	1.69	0.96–2.96	
20000–99999 (n=802)	1.63	0.89–2.99	
100000–199999 (n=390)	0.68	0.32–1.44	
≥200000 (n=1497)	1.59*	1.04–2.41	
Paris area (n=890)	1.07	0.68–1.68	

AOR: adjusted odds ratio.

* p<0.05

** p<0.01

Each odds ratio corresponds to a different logistic regression in which the dependent variable is a quit attempt lasting at least 24 hours in the final quarter of 2016 and the independent variable is ever exposure to the campaign, in the specific subgroup. The models were adjusted for all other characteristics of the table. The interactions between self-reported ever exposure to the campaign and each of the sociodemographic characteristics were tested in separate models, adjusted for all other characteristics of the table. For example, in men, the odds ratio associated with exposure to the campaign was 1.19 (0.92–1.53) (not significant). In women, it was 1.64 (1.14–2.37) (p<0.01). The interaction between gender and exposure to the campaign was not significant (p=0.122). Executive or higher intellectual professionals include senior executives in civil service, corporate administrative and commercial executives, liberal professionals, third-level teachers/professors, engineers, etc. Intermediate professionals include school teachers, middlemanagers, executive officers in the civil service, technicians, etc. Employees include administrative officers in the civil service, administrative or commercial employees, service industry employees, soldiers, etc

## DISCUSSION

### Summary of results and interpretations

This study highlighted an association between exposure to the first edition of the French *Mois sans tabac* smoking cessation campaign in 2016 (selfreported in 2017) and making a QA in the final quarter of that year. For some respondents, these QA lasted beyond the recognized critical withdrawal period of 30 days. Furthermore, a dose–effect relationship between exposure frequency and QA is observed, which is partly explained by the number of sources of campaign exposure. Accordingly, it would appear that the variety of distribution channels used, and the fact that diverse populations were reached, were both success factors for the intervention.

The strength of the association between exposure and QA observed in this indirect analysis is consistent with that measured from the association made by the respondents themselves with the 2016 *Mois sans tabac* intervention: previous analyses estimated 380000 QA linked to the intervention out of a total of 2 million reported QA in France for the last quarter of 2016, corresponding to an increase of 20–25% in the overall number of QA (i.e. related to *Mois sans tabac* or not)^24^.

However, the impact suggested in this study does not take into account the long-term changes in smoking behaviors brought about by an intervention of this size, especially when it is repeated. Nevertheless, the effectiveness of media campaigns would quickly diminish within 2 to 3 months after they end, due to the addictive nature of tobacco and the many socioenvironmental influences that encourage smoking^5,6,25^.

The results according to socioeconomic level were quite mixed. On the one hand, certain priority target populations – specifically blue-collar workers and unemployed workers – seemed to have been less exposed to the campaign, or at least to have a poorer recall of it. On the other hand, the association between exposure to *Mois sans tabac* and QA seems to be greater for unemployed smokers and for women, younger people (aged 18–49 years), smokers with an educational level below high-school diploma, and workers in the French ‘employee’ socio-professional category (see above), even though the interactions were not significant. Previously published preliminary results on the effectiveness of *Mois sans tabac* 2016, based on self-reported associations between QA and the intervention, did not show any difference in the overall impact of socioeconomic status on QA^24^. Beyond the specific relationship with *Mois sans tabac* intervention, the rates of QA in the final quarter of 2016 were higher among those with the highest socioeconomic status (specifically, those with an educational level above high-school diploma and those belonging to the ‘executive or higher intellectual profession’ socio-professional category). This result goes against previous studies conducted in France and elsewhere that indicated that QA was as frequent among people with a low socioeconomic status as in people with a higher status. However, the latter were more likely to succeed^26^. Monitoring of QA according to socioeconomic level should therefore be continued.

The negative association observed in smokers in 2017 between exposure to the campaign and their level of confidence in being able to quit smoking can be interpreted in two ways: smokers declaring themselves very confident about their chances of success if they tried to quit smoking may have paid less attention to public health messages; conversely, people more exposed to these messages but who were not able to quit may have lost confidence in their chance of success. The evaluation of the French nationwide *Messages d’adieux* (Farewell Messages) smoking cessation campaign in 2014, which used emotional messaging and whose content was particularly upsetting, suggested a negative association between exposure to the campaign’s content and confidence in quitting smoking, although no significant association was found after adjusting for confounding factors^22^. According to the literature, it is important to develop messages that reinforce the perceived self-efficacy, especially when it comes to campaigns invoking people’s fears^27^.

A previous study exploring the impact of the *Mois sans tabac* 2016 campaign, and conducted in 2018, showed that among smokers who tried to quit in the final quarter of 2016, whether or not the QA was in connection with the intervention, 6–10% reported being abstinent for at least one year^28^. This is consistent with literature reviews on other interventions which estimated abstinence rates longer than 6 months of 3–5% in the absence of treatment^16,29,30^. In the present study, the use of cessation help services was reported by approximately one in two smokers who tried to quit smoking during *Mois sans tabac* 2016^24^. Furthermore, these long-term abstinence rates did not differ depending on whether the QA in the final quarter of 2016 was attributed to the campaign or not. An evaluation of the *Tips from Former Smokers* (Tips) campaign conducted by the Centers for Disease Control and Prevention (CDC) also showed that the QA success rate among smokers receiving support from a dedicated telephone quitting helpline, was independent from exposure to the Tips campaign^31^.

In France, after a decade of relative stability, the prevalence of daily smoking in the adult population fell sharply between 2016 and 2017, from 29.4 to 26.9%. This unprecedented drop continued in 2018 (25.4%) and 2019 (24.0%)^32,33^. This evolution was the result of several stringent tobacco control measures, some of which were included in France’s 2014–2019 National Tobacco Reduction Program (PNRT)^34^ as follows: 1) an expansion of the list of professionals who can prescribe nicotine substitute treatments, and an increase in reimbursements by the national healthcare system up to 150 euros per year per person for these treatments, both in 2016^35^, with the latter gaining full reimbursement status like other medicines under France’s Health Insurance system in 2018, i.e. without upper limit and with the possibility for the patient not to advance the costs^36^; 2) the implementation of standardized plain packaging in January 2017; and 3) the gradual increase in taxation of tobacco products from 2017 (February 2017 for roll-your-own tobacco and November 2017 for cigarette packs), with the objective that the best-selling packet of 20 cigarettes would cost 10 euros in 2020. At the time of the *Mois sans tabac* 2016 campaign, only a first change in reimbursement of nicotine substitute treatments was fully effective. Thus, the results of the present study on quit attempts made by smokers in the last quarter of 2016 are likely attributable to the campaign itself.

### Strengths and limitations

The present study has several limitations. First of all, a causal relationship between exposure to *Mois sans tabac* and the occurrence of a quit attempt cannot be demonstrated from this type of observational (nonexperimental) design, since it is not possible to control for all systematic or policy implementation factors that could affect both exposure and the outcome itself. This is a common issue in studies that seek to evaluate the effects of communication campaigns^37,38^. Thus, the association measured here might be overestimated, as smokers who were more motivated about stopping smoking or who were attempting to quit may be more likely to notice campaigns that promote smoking cessation. However, in sensitivity analysis on respondents who were still daily smokers at the time of interview in 2017, controlling for importance of quitting and self-confidence in quitting, importance of quitting was not related with selfreported exposure to the campaign and the association between exposure and quit attempt remained. Besides, *Mois sans tabac* campaign benefited from high visibility since 74% of French people aged ≥15 years had heard about it in 2016^39^. The intervention was noteworthy due to the use of mass media as well as a new social marketing approach (branding, call to behavior change, promotion of cessation services etc.) on this topic in France, which could attenuate such recall bias^10^. Additionally, the use of crosssectional retrospective data did not allow assessment of the time order of exposure and outcomes, which is necessary to demonstrate a causal relationship. More generally, memory bias is a possibility, due to the retrospective nature of the study, in particular for individuals interviewed at the end of the survey field (July 2017, i.e. 8 months after the end of the *Mois sans tabac* intervention). Nevertheless, we performed a comparative analysis of self-reported QA rates according to the date of interview and found that the level of memory bias was rather low^24^. Selfreported measure of campaign exposure can lead to false positives, however the utility of confirmed recall compared with aided recall is questioned and a study showed that both recall variables were valid measures of campaign exposure in public health communication evaluations^40^. Finally, most of the analyses were not adjusted for tobacco dependence, as the questions making it possible to characterize dependence could only be put to smokers in 2017, not ex-smokers.

Our study also has several strengths, the first being that it is based on a large representative sample of the population residing in mainland France. The participation rate (48.5%) is of the same order as that observed in main health behavior surveillance surveys such as the US Behavioral Risk Factor Surveillance System Survey (BRFSS)^41^, and poststratification weighting was used to correct for main sociodemographic variables on the French population structure. Second, the fact that the whole of the final quarter of 2016 was considered allows to partly control for any ‘windfall’ effect, whereby smokers who would have made a QA in October or December in the absence of *Mois sans tabac* decided to switch the month of their attempt to November.

## CONCLUSIONS

The present study’s results suggest the effectiveness of the first edition of the *Mois sans tabac* campaign on quit attempts. Those results are similar to those observed in the evaluation of the first edition of the British *Stoptober* campaign, based on a different design that was monthly cross-sectional surveys on quit attempts^9^. Finally, this study provides an overall view of quit attempts made by smokers during the *Mois sans tabac* social marketing intervention in 2016, irrespective of the modalities they chose to achieve it, i.e. whether or not they had chosen to be accompanied, to register online for the operation, or to use treatments to help them quit smoking.

## Data Availability

The data supporting this research is available from the authors on reasonable request.
