# Supplementary material for: Effectiveness of ‘Mois sans tabac 2016’: A French social marketing campaign against smoking
Source: Tob Induc Dis. 2021 Jul 19;19:60. doi: 10.18332/tid/139028 (PMC8288465; doi:10.18332/tid/139028)
Supplement: Supplementary file 1 [file TID-19-60-s1.pdf]

Table S1: Factors associated with ever exposure to *Mois sans tabac*, among daily smokers at the time of the interview in 2017: socio-demographic factors, tobacco dependence, importance of quitting smoking and self-confidence in quitting (N = 5,968)

	%	aOR	95%CI
**Sex**	***		
Men (ref.) (n=3,078)	79.2	1	
Women (n=2,890)	87.3	1.55***	[1.32-1.83]
**Age**	***		
18-24 years old (ref.) (n=675)	77.6	1	
25-49 years old (n=3,153)	80.7	1.19	[0.94-1.51]
50 years old and over (n=2,140)	89.5	2.27***	[1.75-2.94]
**Educational level**			
< High-school diploma (ref.) (n=2,649)	82.3	1	
High-school diploma (n=1,428)	83.9	1.21	[0.99-1.48]
> High-school diploma (n=1,880)	84.2	1.15	[0.93-1.42]
**Professional status**	***		
Working (ref.) (n=3,704)	83.4	1	
Unemployed looking for a job (n=721)	75.6	0.66***	[0.54-0.82]
Inactive (n=1,543)	86.1	1.19	[0.96-1.47]
**Socio-professional category**	***		
Farmer, craftsperson, trader or business manager (independent professional) (n=465)	77.9	0.60***	[0.45-0.81]
Executive or higher intellectual professional (n=726)	82.0	0.73*	[0.56-0.94]
Intermediate professional (ref.) (n=1,506)	87.1	1	
Employee (n=1,671)	85.4	0.93	[0.74-1.17]
Blue-collar worker (n=1,541)	80.2	0.78*	[0.61-0.98]
**Size of area of residence**	***		
Rural (ref.) (n=1,532)	88.4	1	
< 20,000 inhabitants (n=1,060)	85.3	0.82	[0.64-1.04]
20,000 – 99,999 inhabitants (n=755)	79.6	0.67**	[0.52-0.88]
100,000 – 199,999 inhabitants (n=361)	80.5	0.69*	[0.49-0.97]
>= 200,000 inhabitants (n=1,422)	82.0	0.73**	[0.58-0.91]
Paris area (n=825)	77.0	0.50***	[0.39-0.64]
**Heaviness of smoking index**	*		
0-1 (ref.) (n=2,756)	82.1	1	
2-3 (n=2,228)	85.3	1.30**	[1.09-1.54]
4-6 (n=933)	80.6	0.93	[0.74-1.16]
**Importance of quitting smoking (continuous, 0-10)**		1.01	[0.99-1.04]
**Self-confidence in quitting (continuous, 0-10)**		0.95***	[0.92-0.98]

aOR : adjusted odds-ratio, CI : confidence interval. *: p<0.05; **: p<0.01; ***: p<0.001

Note: Percentages of respondents who recalled *Mois sans tabac* and p-values for bivariate analyses between sociodemographic variables and recall of *Mois sans tabac*, adjusted odds-ratios from logistic regression including all variables in the table.

Executive or higher intellectual professionals include senior executives in civil service, corporate administrative and commercial executives, liberal professionals, third-level teachers/professors, engineers, etc. Intermediate professionals include school teachers, middle-managers, executive officers

in the civil service, technicians, etc. Employees include administrative officers in the civil service, administrative or commercial employees, service industry employees, soldiers, etc.

Table S2: Factors associated with 24-hour quit attempt in the final quarter of 2016, among daily smokers at the time of the interview in 2017: socio-demographic factors, exposure to the campaign, tobacco dependence, importance of quitting smoking and self-confidence in quitting (N = 5,968)

	%	aOR	95%CI
**Sex**			
Men (ref.) (n=3,078)	14.3	1	
Women (n=2,890)	13.3	0.97	[0.82-1.15]
**Age**	***		
18-24 years old (ref.) (n=675)	22.8	1	
25-49 years old (n=3,153)	12.9	0.46***	[0.36-0.58]
50 years old and over (n=2,140)	11.6	0.50***	[0.39-0.63]
**Educational level**	**		
< High-school diploma (ref.) (n=2,649)	12.2	1	
High-school diploma (n=1,428)	15.5	1.04	[0.85-1.28]
> High-school diploma (n=1,880)	16.3	1.14	[0.92-1.41]
**Professional status**			
Working (ref.) (n=3,704)	13.1	1	
Unemployed looking for a job (n=721)	16.0	1.15	[0.90-1.46]
Inactive (n=1,543)	14.4	0.99	[0.81-1.21]
**Socio-professional category**	**		
Farmer, craftsperson, trader or business manager (independent professional) (n=465)	10.8	0.85	[0.61-1.19]
Executive or higher intellectual professional (n=726)	19.7	1.19	[0.93-1.53]
Intermediate professional (ref.) (n=1,506)	15.0	1	
Employee (n=1,671)	12.4	0.87	[0.70-1.09]
Blue-collar worker (n=1,541)	13.4	0.96	[0.75-1.22]
**Size of area of residence**			
Rural (ref.) (n=1,532)	11.8	1	
< 20,000 inhabitants (n=1,060)	12.5	0.95	[0.75-1.22]
20,000 – 99,999 inhabitants (n=755)	12.6	1.09	[0.83-1.42]
100,000 – 199,999 inhabitants (n=361)	15.5	1.17	[0.83-1.64]
>= 200,000 inhabitants (n=1,422)	15.9	1.18	[0.95-1.46]
Paris area (n=825)	15.4	0.94	[0.72-1.22]
**Heaviness of smoking index**			
0-1 (ref.) (n=2,756)	19.1	1	
2-3 (n=2,228)	10.4	0.51***	[0.43-0.61]
4-6 (n=933)	8.4	0.46***	[0.35-0.60]
**Importance of quitting smoking (continuous, 0-10)**		1.14***	[1.11-1.18]
**Self-confidence in quitting (continuous, 0-10)**		1.06***	[1.03-1.09]
**Had heard of *Mois sans tabac*[a]**			
No (ref.) (n=900)	13.0	1	
Yes (n=5,068)	14.0	1.26*	[1.01-1.59]

**Frequency of exposure to *Mois sans tabac*[a]**			
Never (ref.) (n=900)	13.0	1	
Less than weekly (n=570)	11.3	0.99	[0.71-1.39]
Weekly (n=1,935)	15.2	1.24	[0.97-1.59]
Daily (n=2,394)	13.9	1.39**	[1.09-1.77]

aOR : adjusted odds-ratio, CI : confidence interval. *: p<0.05; **: p<0.01; ***: p<0.001

Note: Percentages of quit attempts and p-values for bivariate analyses between sociodemographic variables and quit attempts, adjusted odds-ratios from logistic regressions.
Executive or higher intellectual professionals include senior executives in civil service, corporate administrative and commercial executives, liberal professionals, third-level teachers/professors, engineers, etc. Intermediate professionals include school teachers, middle-managers, executive officers in the civil service, technicians, etc. Employees include administrative officers in the civil service, administrative or commercial employees, service industry employees, soldiers, etc.

[a] Results of the logistic model after adjustment for sex, age, educational level, professional status, socio-professional category, size of the area of residence, tobacco dependence (Heaviness of smoking index), importance of quitting and self-confidence in quitting.

© 2021 Guignard R. et al.
